# A Prognostic Tool for Individualized Prediction of Graft Failure Risk within Ten Years after Kidney Transplantation

**DOI:** 10.1155/2019/7245142

**Published:** 2019-04-08

**Authors:** Danko Stamenic, Annick Rousseau, Marie Essig, Philippe Gatault, Mathias Buchler, Matthieu Filloux, Pierre Marquet, Aurélie Prémaud

**Affiliations:** ^1^INSERM, U1248, F-87000 Limoges, France; ^2^University of Limoges, UMR_1248, F-87000 Limoges, France; ^3^CHU Tours, Service Néphrologie et Immunologie Clinique, F-37000 Tours, France; ^4^CHU Limoges, Service d'immunologie et immunogénétique, F-87000 Limoges, France; ^5^CNRS, CRIBL, UMR 7276, F-87000 Limoges, France; ^6^CHU Limoges, Service de Pharmacologie, Toxicologie, et Pharmacovigilance, F-87000 Limoges, France

## Abstract

Identification of patients at risk of kidney graft loss relies on early individual prediction of graft failure. Data from 616 kidney transplant recipients with a follow-up of at least one year were retrospectively studied. A joint latent class model investigating the impact of serum creatinine (Scr) time-trajectories and onset of* de novo *donor-specific anti-HLA antibody (*dn*DSA) on graft survival was developed. The capacity of the model to calculate individual predicted probabilities of graft failure over time was evaluated in 80 independent patients. The model classified the patients in three latent classes with significantly different Scr time profiles and different graft survivals. Donor age contributed to explaining latent class membership. In addition to the SCr classes, the other variables retained in the survival model were proteinuria measured one-year after transplantation (HR=2.4, p=0.01), pretransplant non-donor-specific antibodies (HR=3.3, p<0.001), and* dn*DSA in patient who experienced acute rejection (HR=15.9, p=0.02). In the validation dataset, individual predictions of graft failure risk provided good predictive performances (sensitivity, specificity, and overall accuracy of graft failure prediction at ten years were 77.7%, 95.8%, and 85%, resp.) for the 60 patients who had not developed* dn*DSA. For patients with* dn*DSA individual risk of graft failure was not predicted with a so good performance.

## 1. Introduction

In kidney transplantation, a new challenge in modelling is individualized prediction of graft failure risk over time. Up to now, no study has reported such a model appropriate for any kidney transplant patients to assess the individual risk and its evolution with time. Numerous risk factors of kidney graft failure are known: factors linked to donor (e.g., age, cause of death, serum creatinine, living or deceased donor, cause of death, and Expanded Criteria Donor, ECD) [1–6], to transplantation (e.g., cold ischemia time and retransplantation) [7], and to recipients (demographic, clinical, immunological, and biological factors) [8–14]. Several recent studies have identified donor-specific anti-HLA antibodies (DSA) and antibody-mediated rejection (ABMR) as primary causes of allograft failure [9, 15–17]. Ways to improve graft survival in patients who do not develop DSA are less studied although many graft failures are observed in patients without DSA [18].

Association between graft failure and serum creatinine (SCr) was studied in taking into account SCr levels measured at specific time-points and/or SCr linear evolution with time after transplantation (e.g., SCr slopes between two measurements) [5, 14]. Considering the whole dynamic history of SCr (i.e., SCr evolution with time) should be an efficient alternative strategy.

Latent class models could permit studying the heterogeneity in the individual time-trajectories of SCr [19]. The joint models are innovative statistical tools which allow studying the association between evolution of markers over time (i.e., time-trajectories of continuous variable), fixed covariates (i.e., individual factors collected at a given time), and onset of an event [5, 20]. Statistical developments in the joint modelling area rely either on the shared random effects models that include characteristics of the longitudinal marker as predictors in the model for the time-to-event [21, 22] or on the joint latent class models which assume that the population can be parted into homogeneous subgroups (corresponding to latent classes), with a class-specific time-evolution of the marker and a class-specific risk of the event [23, 24]. Using a shared random effect model, Fournier* et al. *(2016) showed that the risk of graft failure up to 13 years after transplantation was associated with both current value and current slope of SCr [5]. Onset of* de novo* DSA (*dn*DSA) was not considered by the authors and no prediction of the individual graft failure risk was obtained. No joint latent class model has been developed previously to predict graft failure.

As several papers reported models predictive of graft failure using data collected up to one year after transplantation, it seemed relevant (i) to jointly model the change of serum creatinine over at least the first year after transplantation and (ii) to investigate in such a model the impact of individual potential risk factors on both change of SCr and graft failure risk. Therefore, the objectives of the present study were (i) to develop a joint latent class model investigating the impact of serum creatinine time-trajectories and onset of* dn*DSA on graft survival and (ii) to study the possibility of individualized risk prediction of kidney graft failure within ten years after transplantation.

## 2. Material and Methods

### 2.1. Study Population

**Figure 1:**
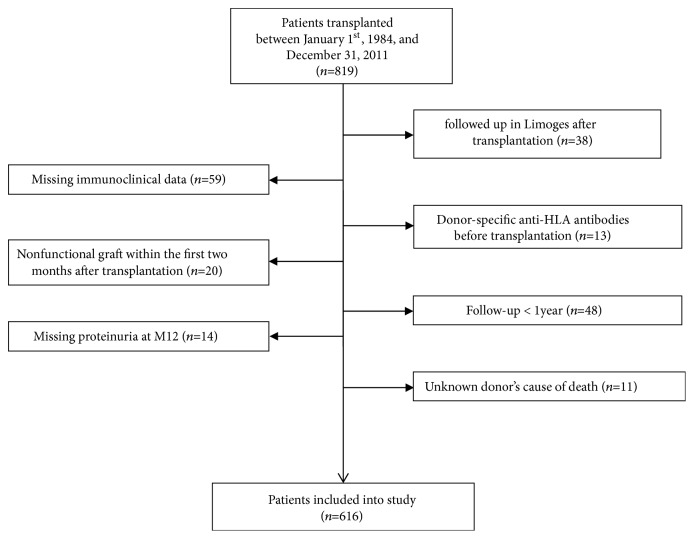
Flowchart showing selection of renal transplant recipients included in the study.

Data was extracted from the retrospective cohort of kidney transplant recipients grafted at the University Hospital of Limoges (France) between 1984 and the end of 2011 (n=819). Among these patients, 616 who had sufficient data with a clinical and immunological follow-up of at least one year were included in the study. A flowchart showing patient selection is presented in Figure 1.

The study database was approved by the French Informatics and Liberty National Commission (CNIL, registration number 1795293). All the grafts came from heart-beating deceased donor. More details about the patients included can be found in a previous work of our group [10].

### 2.2. Outcomes and Study Endpoint

Graft failure, defined as return to dialysis or preemptive retransplantation, was used as the outcome variable. Death was considered as a censored event when the recipient died with a functioning graft.

### 2.3. Available Variables

Donor-specific variables were age and cause of death (categorized to vascular, traumatic vehicle accident, traumatic nonvehicle accident, and others). Transplantation-related variables included cold ischemia time, retransplantation, and transplantation period (1984-1993, 1994-2002, and 2003-2011). Recipient variables included the following: age at transplantation, gender, nondonor-specific anti-human leucocyte antigen antibodies (NDSA) before transplantation, initial immunosuppressive regimen, and proteinuria levels at month 12 (M12) after transplantation (in case of missing data for proteinuria at month 12, the first value collected between M12 and M18 was used). Additionally, repeated measures of SCr within the first 18 months after transplantation (usually at M1, M3, M6, M12, and M18, median number of measurements: 5, range: 2-8), diagnosis of the first acute rejection episode (AR), and onset of* de novo* donor specific anti-HLA antibodies (*dn*DSA) were collected.

Anti-HLA antibodies were screened and identified using Luminex® solid-phase assay (One Lambda LABScreen assays) in samples collected before transplantation, at three, six, and twelve months after transplantation and annually thereafter or whenever clinically indicated. Results were expressed as median fluorescence intensity (MFI). MFI>1000 was considered positive. All sera collected and tested using the Complement Dependent Cytotoxicity method prior to availability of Luminex® technology in our center (2007) were reanalyzed using Luminex® as previously described.[10] Patients in whom the Luminex® reanalysis identified presence of DSA before transplantation were excluded from the database studied.

Donor, recipient, and transplant characteristics are presented in Table 1.

### 2.4. Statistical Analysis

#### 2.4.1. Joint Latent Class Model

A joint latent class model for a longitudinal outcome and a right-censored (left-truncated) time-to-event outcome was developed in the “lcmm” R-Package, version 17.8 (available at https://cran.r-project.org/web/packages/lcmm/lcmm.pdf). This model considers the population of subjects as heterogeneous and assumes that the population consists of a finite number of homogeneous subgroups (the so-called latent classes)[24, 25]. Each latent class was characterized by a class-specific time-trajectory of SCr and a class-specific risk of graft failure. This type of joint model constitutes of three submodels: (1) a multinomial logistic submodel aiming at calculating each patient probability of belonging to each latent class, (2) a mixed effect submodel to describe the SCr time-trajectories specific of each class, and (3) a survival submodel to describe the risk of graft failure specific of each class. A general mathematical representation of these submodels, as well as R codes, can be found elsewhere [19, 25].

The model was constructed in a step-by-step procedure. The first step of model building aimed at defining (i) a mixed-effects model for the SCr trajectories, (ii) the baseline risk function, and (iii) the number of latent classes. Different link functions were compared to transform the observed SCr values into a Gaussian latent variable (i.e., herein, the unobserved kidney function): (i) a linear transformation, (ii) a rescaled cumulative distribution function of a beta distribution, (iii) quadratic I-splines with equidistant nodes, and (iv) quadratic I-splines with nodes located at the quantiles of SCr distribution. The most appropriate link function was selected on the basis of goodness-of-fit as measured by the discretized Akaike criterion (dAIC) [25]. The risk of graft failure was modelled using a parametric proportional-hazards model. Weibull, piecewise constant, and M-splines baseline risk functions were tested and compared using the Akaike criterion (AIC). The joint latent class model was estimated for a number of latent classes varying from 1 to 5 and the Bayesian information criterion (BIC) was used to compare them [25].

In the second step, the impact of the available covariates (see Table 1) as well as the impact of their interaction on (i) the class-membership probabilities and (ii) both class-specific SCr trajectories and graft failure risk was studied through fixed effects common within all classes and/or class-specific effects. Each covariate was first tested in univariate analysis and entered in multivariate analysis when univariate association (p<0.2) was found. If the onset of* dn*DSA was retained as covariate, its impact would be studied by taking into account several post-*dn*DSA follow-up periods because associated adverse effects are known to be delayed from their onset. The criteria for final model selection were the BIC and the highest mean posterior class membership probabilities which assess the ability of the model to discriminate between the different latent classes. Finally, the predicted class-specific survivals were compared with the observed survivals within each class using Kaplan-Meier analysis.

Because certain research teams studied the factors predictive of short-term graft survival [9], we also analyzed the factors predictive of 5-year graft survival. Numerous studies having investigated the predictive factors of graft failure among the individual factors known up to one year after transplantation, the final joint model was compared to a model including follow-up data collected up to 1 year after transplantation only.

#### 2.4.2. Individual Predictions in an Independent Patient Group

An independent database of 80 patients (60 without and 20 with* dn*DSA randomly selected) grafted since 2002 and followed up in another French transplant center (CHU Tours, Aster database approved by the CNIL, authorization number DR-2012-518) was used to evaluate the capacity of the model to calculate individual predicted probabilities of graft failure over time [26].

## 3. Results

### 3.1. Follow-Up Description

Among the 616 patients studied, graft failure was observed in 68 (11%) patients over the 10 years of follow-up (incidence per 1,000 person-years, 16.8; 95% CI, 13.1 to 21.3). The median follow-up time in patients up to graft failure was 4.97 years (range: 1-10). Among 548 event-free patients, median follow-up time was 7.13 years (range: 1-10). There were 56 deaths with a functional graft. Sixty patients developed* dn*DSA (incidence per 1 000 person-years, 14.8; 95% CI, 11.3 to 19.1; median time of onset 3.93 years; range: 0.02-9.8) and 12 (20%) of them lost their graft. In these 60 patients, the median follow-up time up to graft failure was 6.13 years (range: 1-10). In the 556 patients who did not develop* dn*DSA, graft failure was observed in 56 patients (11.2%) over the 10 years of follow-up. The median follow-up time to graft failure in these 556 patients was 4.39 years (range: 0.94-10). One hundred and thirty-five patients were treated for the first acute rejection episode over the whole study period, 121 (90%) of which were biopsy proven. T-cell mediated rejection (TCMR) was evidenced in 104 patients, ABMR in 14 patients, and mixed rejection (TCMR+ABMR) in 3 patients. Ninety-four first rejections occurred within the first year after transplantation.

### 3.2. Joint Latent Class Modelling

The SCr time-trajectories were fitted after transformation with a I-spline link function with 5 equidistant nodes since it provided the lowest dAIC. The time-trajectories of SCr after transformation were best described using quadratic function of time to allow nonlinear mean trajectories over time. The baseline risk function was modelled parametrically using a two-parameter Weibull baseline risk function. The joint latent class model including three latent classes was retained. The class-specific risks of graft failure were described using presence of NDSA before transplantation, proteinuria at M12 greater than 0.275 g/L (yes/no), and interaction between onset of acute rejection and development of* dn*DSA (yes/no). The estimations related to the proportional hazard submodel of the final joint model are reported in Table 2.

The donor age (categorized as greater or not greater than 60 years) contributed to explaining latent class membership with the recipients of kidneys from donors younger than 60 years having a significantly higher probability to be allocated to class 1 (characterized by the lowest Scr values and the best graft survival). The mean posterior probability of belonging to each class ranged from 82.6% in patients allocated to class 1 to 89.2% in class 3, indicating a clear discrimination between the latent classes. Of note, this model including acute rejection and* dn*DSA data collected over the follow-up outperformed a joint model taking into account data collected up to the end of the first year after transplantation only (p=0.001). This comparison showed the added value of the* dn*DSA data collected after one year after transplantation.

**Figure 2:**
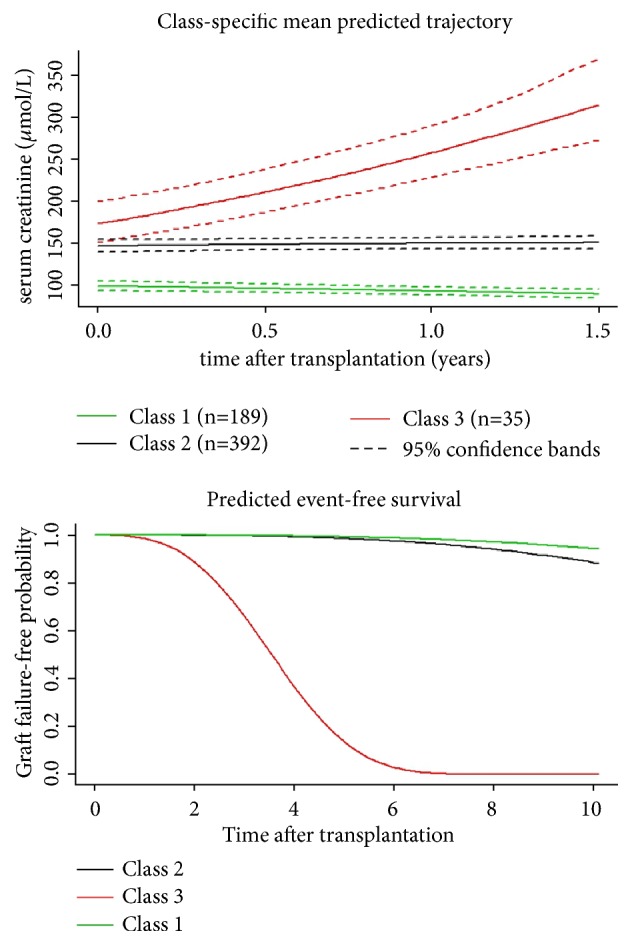
Class-specific predicted mean trajectories (top panel) and class-specific predicted event-free probabilities (bottom panel) from the final joint latent-class mixed model; class 1 (n=189) is in green, class 2 (n=392) in black, and class 3 (n=35) in red. Dashed lines are the computed 95% confidence intervals.

Figure 2 shows the estimated trajectories retranslated into SCr and the associated predicted graft failure-free survival for each class. Class 1 with 189 patients (30.7%) was characterized by a mean SCr baseline value close to 100 *μ*mol/L, a slow decrease in SCr within the first 18 months after transplantation, and a mean risk of graft failure at 10 years after transplantation close to 5%. Class 2 corresponding to the majority of the patients (n=392, 63.6%) was characterized by a higher mean SCr baseline value close to 150 *μ*mol/L and a stable mean trajectory over the first 18 months after transplantation while the mean risk of graft failure at 10 years after transplantation achieved 10%. In comparison with class 1, it was associated with a significant increase in the observed incidence of graft failure at 10 years after transplantation (log-rank test, p=0.0346). Finally, class 3 with 35 patients (5.7%) was characterized by a mean SCr value close to that of class 2 at baseline followed by a rapid rise of SCr within the first 18 months after transplantation. In comparison with class 1 and class 2, it was associated with a significant increase in the observed incidence of graft failure (p<0.0001). The mean risk of graft failure at 10 years after transplantation in this class was 100%, and no subject in this class had a graft survival greater than seven years.

The short-term risk (5 years) of graft failure was also studied using the developed joint three latent classes' model. This 5-year risk was significantly associated with serum creatinine latent classes (p<0.0001), proteinuria at M12 (p=0.003), and pretransplant NDSA (p=0.034). Contrary to the 10-year model, the effect of interaction between* dn*DSA and acute rejection was not significant any more.

### 3.3. Individual Predictions in an Independent Patient Group

**Figure 3:**
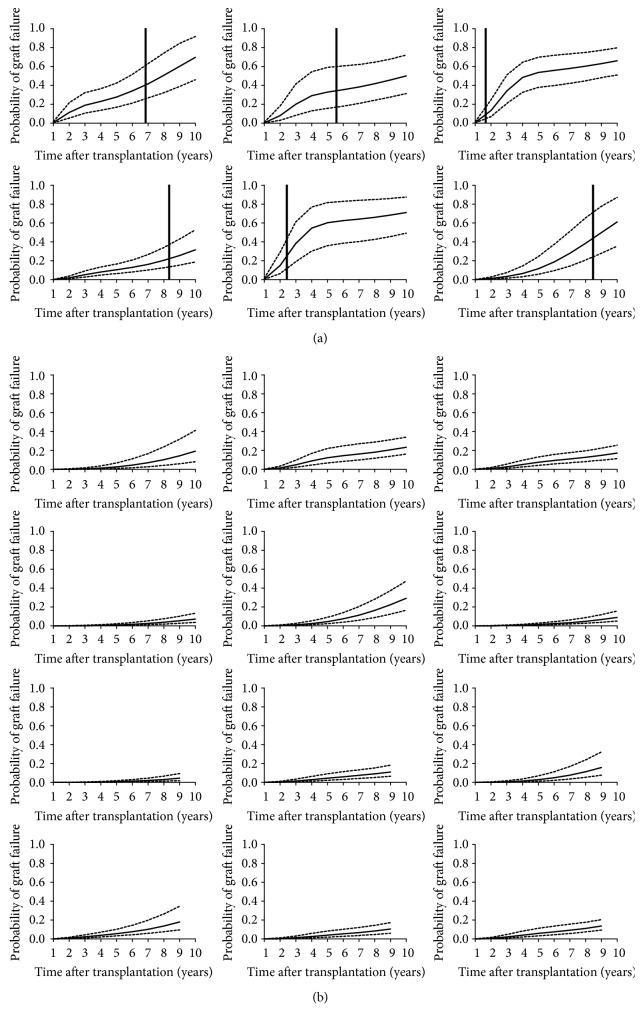
Individual predictions of 10-year graft failure risk based on covariates known at 1 year after transplantation for 18 patients without* dn*DSA (a) who experienced graft failure and (b) who did not experience graft failure. Solid lines indicate the predicted medians and dashed lines indicate the 95% confidence intervals; the vertical line indicates time of graft failure.

Individual predictions of graft failure up to the end of follow-up were computed for 60 patients from the validation dataset who had not developed DSA, according to their observed history of SCr and the covariates retained in the final 10-year joint model. In the 36 tested patients with graft failure, failure risk was adequately predicted in 28 patients as the 95% confidence interval of the predicted probability of graft failure included values greater than 50%. In the 24 patients who did not experience graft failure, the predicted probability of graft failure remained lower than 30% (with an upper limit of the 95% confidence interval<50%) until the end of the follow-up except for one patient. Thus, using data collected up 12 months after transplantation in this patient subpopulation, sensitivity, specificity, and overall accuracy of the graft failure prediction at ten years were 77.7%, 95.8%, and 85%, respectively.  Figure 3 depicts the predicted probability of graft failure in 18 patients randomly selected from this subgroup.

**Figure 4:**
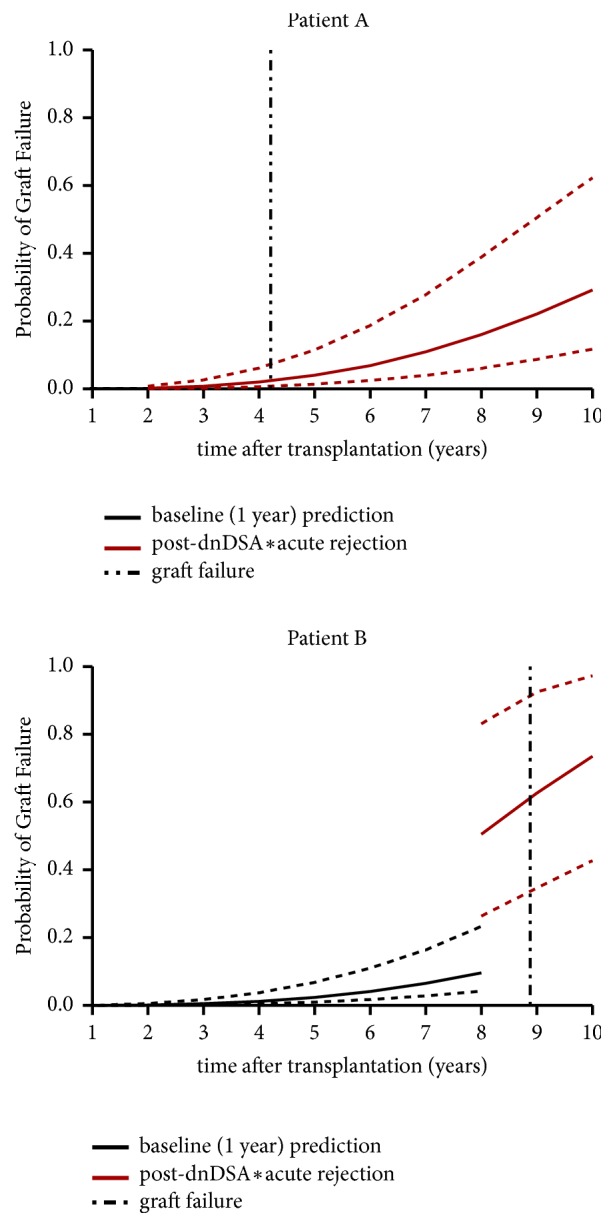
The worst (patient A) and the best (patient B) prediction of probabilities of 10-year graft failure with 95% confidence intervals from the final joint latent-class mixed model among the group of patients who developed* de novo *donor-specific anti-HLA antibody (*dn*DSA). The black part of the curve corresponds to predictions based on covariates known up to 1 year after transplantation while the red curve corresponds to prediction recalculated after onset of both* dn*DSA and acute rejection; the vertical dashed black line indicates the time of graft failure.

In the 20 tested patients who had developed* dn*DSA, the model predicted an increased risk of graft failure, but the individual risk of graft failure was not adequately predicted for most of these patients. The best and worth predicted curves of graft failure obtained in this patient subgroup are shown in Figure 4.

## 4. Discussion

This study presents a new tool which adequately predicts the individual risk of graft failure in patients who did not develop* dn*DSA. In patients with* dn*DSA, individual prediction of graft failure risk was not obtained with a so good accuracy. The variables retained in the model are patient variables routinely collected and are classically reported to be associated with graft failure (measurements of SCr and proteinuria, presence of pretransplant NDSA,* dn*DSA, acute rejection, and donor age) [8–10, 12, 14, 27]. Our study confirms the association of donor age above sixty years with both worse renal function and shorter graft survival [6, 28]. In the model developed herein, the proteinuria level observed at one year after transplantation also contributed to explaining the graft failure risk. Proteinuria at M12 was previously retained in association with several SCr values determined within the first year after transplantation in the KTFS score aiming at predicting the graft survival at 8 years [14]. Our model included an interaction term between* dn*DSA and acute rejection showing, as previously reported, that* dn*DSA are more deleterious for graft survival when the patient has also experienced acute rejection [11]. This work confirms the deleterious impact of pretransplant NDSA which was less studied than the impact of preformed DSA but was found to influence clinical decisions in personalized medicine [29, 30].

Although numerous works highlighted the potential impact of certain DSA classes on graft failure [9, 10, 31], nearly all reported survival models and scoring systems developed to predict graft survival in the kidney-transplant population did not take into account the onset of* dn*DSA [5, 8, 14]. At side, some studies focused on patients with preexisting and/or* de novo* DSA [17]. Recently, Viglietti et al. reported a new score to predict kidney allograft survival in patients with preexisting or* de novo* DSA and who experienced ABMR [31]. Ignoring the impact of* dn*DSA on the prediction of graft failure risk in a predictive tool could lead not only to underestimating this risk in patients with* dn*DSA but also to overestimating the risk in patients without* dn*DSA, especially in the long term. Herein, taking into account* dn*DSA it improved on average the long-term survival prediction but not the short-term one (e.g., 5-year graft survival). Consistently, Gonzales et al. found that adding presence of* dn*DSA at 1 year after transplantation to an existing risk model (which incorporates recipient factors at 1 year, including age, sex, ethnicity, renal function, proteinuria, and acute rejection) [8] did not improve predictive ability of graft loss by 5 years [9]. This result could be due to a too short time horizon because (i)* dn*DSA occur all over the follow-up and are mostly absent in the first year after transplantation and (ii) graft loss attributable to* dn*DSA can occur several years after their onset [12]. Recently, significant progress has been made to understand the pathophysiology of DSA-mediated injuries and the determinants of graft loss [17, 32, 33].

Preliminary tests were performed from our model for making individualized risk predictions in distinguishing patients with and without* dn*DSA.

The graft failure risk has been less studied in patients who had not developed* dn*DSA. However, most of the kidney graft failures are observed in this subpopulation. The frequency of graft failure observed herein (in database used for model development) was similar to the frequency reported by Terasaki's team (11% allograft loss) with a similar follow-up (median of 94 months) [18]. In this population, predictive performance of our model seems high. Using the validation dataset, graft loss was actually observed in 28 out of the 29 patients without* dn*DSA for whom the graft failure was predicted by our model (i.e., one false positive). As a comparison, a sensitivity of 0.72 and a specificity of 0.71 were reported for the Kidney Transplant Failure Score [14]. Although our model might not be appropriate for predictions of graft loss in the global kidney transplant population, it can still be used to generate more than satisfying individual predictions in the majority of this population (i.e., the patients who do not develop* dn*DSA). It is noteworthy that this prediction can be performed for one year after transplantation using data routinely collected in clinical setting. Interestingly, this prediction tool does not require histologic data, which is in accordance with the current practice to decrease the use of biopsies.

Great differences between the present model and the previously published tools for graft failure prediction are in (i) predicting the individual risk of graft failure over time contrary to scoring systems which classified the patient in a risk class (e.g., 3- or 4-level system) [10, 14, 31] and (ii) taking into account the time-evolution of Scr levels within the first year after transplantation contrary to works which consider single time-points [14].

We used for the first time the recently proposed statistical approach of joint latent class models to predict graft outcome. Interestingly, the strengths of this approach have been demonstrated in oncology [34] and dementia [23].

While we are in an era with very few new therapeutic strategies and new immunosuppressive drugs, individual prognostic tools are necessary for the optimal selection of patients in clinical trials. To demonstrate significant effects of candidate molecules, future trials should focus on patients with poor renal prognoses, and we believe that our model may be a valuable tool for identification of these patients.

Last, our findings should be interpreted by taking into account the limitations of current study. We were unable to directly test the impact of immunosuppressive regimens and their blood levels because of dose adjustments and switches from one regimen to another which occurred frequently in patients over such a long study period (from 1984 until 2011). However, we would expect that the different immunosuppressive regimens are at least in part related to different transplantation period, and the period of transplantation was tested but not among the covariates significant in the multivariate model. Similarly, two out of four criteria for expended donation (i.e., last donor SCr and history of hypertension) were missing in the present study but by combining the two remaining criteria in a single dichotomous variable (i.e., donor age ≥60 years or between 50 and 59 years with cardiovascular accident vs. others) we did not observe a better performance of our model than using donor age alone.

Although allograft histology thanks to repeated biopsies also was found to be associated with transplant outcome [31], it was not possible to investigate its impact in the present study. Indeed, the database included almost exclusive biopsies performed when there were clinical signs in favour of graft lesions, such as an increase in serum creatinine. Anyway, the purpose of this work was to develop a simple-to-use tool taking into account routinely collected data after transplantation. This is in accordance with the general trend to decrease the graft biopsy appeal.

## 5. Conclusion

Joint models were used to characterize the kinetics of Scr and their link with time-to-event (time-to-graft failure) and to identify relevant covariates linked to graft survival. The individual predictions of graft failure probability obtained in patients without DSA show that this approach could be useful to improve patient's follow-up and the early detection of numerous at-risk patients as approximately half of graft failures are observed in patients without DSA. The graft failure risk would be reevaluated throughout the time after transplantation in case of dnDSA occurrence or acute rejection. In the future, we have the project to include our predictive model in an expert system available for transplant physicians.

## Figures and Tables

**Table 1:** Immunological parameters, donor, recipient, and transplant characteristics (n=616).

Donor characteristics	
Mean age (SD) [years]	43.5 (16.4)
Age ≥60 years (n)	110 (17.8%)
Cause of death (n)	
*Vascular*	268 (43.5%)
*Traumatic vehicle accident*	106 (17.2%)
*Traumatic nonvehicle accident*	116 (18.8%)
*Others*	36 (5.8%)
*Unknown*	90 (14.6%)

Recipient characteristics	

Age (years, mean (SD))	49.5 (13.8)
Male/female (n)	375/241

Biological parameters	

Mean proteinuria measured at M12 [g/L] (SD)	0.166 (0.451)
Mean serum creatinine at month 12 [*μ*mol/L] (SD)	139 (67)

Clinical characteristics	

Death with functioning graft (n)	56 (9.1%)
Acute rejection (n)	135 (21.9%)
Graft failure (n)	68 (11.0%)

Initial immunosuppressant	

AZA/MMF	134/473
*Unknown*	9

Immunological parameters	

*De novo* donor specific anti-HLA antibodies (n)	60 (9.7%)
Non–donor-specific anti-HLA before transplantation (n)	96 (15.6%)

Transplant characteristics	

Retransplantation (n)	52 (8.5%)
Mean cold ischemia time [minutes] (SD)	1138 (369)
Period of transplantation (n)	
*From 1984 to the end of 1993 *	99 (16.0%)
*From 1994 to the end of 2002 *	194 (31.5%)
*From 2003 to the end of 2011 *	323 (52.5%)

AZA = azathioprine; MMF = mycophenolate mofetil.

**Table 2:** Joint latent class mixed model estimates of hazard ratio for graft failure risk.

	Survival submodel		
	HR	95% CI	p-value
NDSA before transplantation (yes vs. no)	3.27	[1.75 - 6.13]	<0.001
Proteinuria at M12 (>0.275 g/L vs. ≤0.275 g/L)	2.41	[1.22 - 4.76]	0.011
*dn*DSA (yes vs. no)	0.49	[0.05 - 4.46]	0.524
Acute rejection (yes vs. no)	0.78	[0.39 - 1.56]	0.486
Interaction* (dn*DSA*∗*acute rejection)	15.35	[1.55 -152.43]	0.019

HR= hazard ratio, CI= confidence interval, NDSA=non-donor-specific anti-HLA antibodies, and *dn*DSA=*de novo* donor-specific anti-HLA antibodies.

## Data Availability

The relevant data used to support the findings of this study are within the article.
